# Use of an Ecosystem-Based Approach to Shed Light on the Heterogeneity of the Contamination Pattern of *Listeria monocytogenes* on Conveyor Belt Surfaces in a Swine Slaughterhouse in the Province of Quebec, Canada

**DOI:** 10.3390/pathogens10111368

**Published:** 2021-10-22

**Authors:** Fanie Shedleur-Bourguignon, William P. Thériault, Jessie Longpré, Alexandre Thibodeau, Philippe Fravalo

**Affiliations:** 1 NSERC Industrial Research Chair in Meat Safety (CRSV), Faculté de Médecine Vétérinaire, Université de Montréal, Saint-Hyacinthe, QC J2S 2M2, Canada; fanie.shedleur-bourguignon@umontreal.ca (F.S.-B.); william.p.theriault@umontreal.ca (W.P.T.); alexandre.thibodeau@umontreal.ca (A.T.); 2 F. Ménard, Division d’Olymel s.e.c., Ange-Gardien, QC J0E 1E0, Canada; JessieLongpre@olymel.com; 3 CRIPA Swine and Poultry Infectious Diseases Research Center, Faculté de Médecine Vétérinaire, Université de Montréal, Saint-Hyacinthe, QC J2S 2M2, Canada; 4 Pôle Agroalimentaire, Conservatoire National des Arts et Métiers (Cnam), 75003 Paris, France

**Keywords:** *Listeria monocytogenes*, accompanying microbiota, conveyor belt surfaces, heterogeneous spatial contamination, *Veillonella*

## Abstract

The role of the accompanying microbiota in the presence of *Listeria monocytogenes* on meat processing surfaces is not yet understood, especially in industrial production conditions. In this study, 300 conveyor belt samples from the cutting room of a swine slaughterhouse were collected during production. The samples were subjected to the detection of *L. monocytogenes*. Recovered strains were characterized by serogrouping-PCR, InlA Sanger sequencing and for their ability to form biofilm. A selection of isolates was compared with core genome multi-locus sequence typing analysis (cgMLST). The sequencing of the V4 region of the 16S RNA gene of the microorganisms harvested from each sample was carried out in parallel using the Illumina MiSeq platform. Diversity analyses were performed and MaAsLin analysis was used to assess the link between *L. monocytogenes* detection and the surrounding bacteria. The 72 isolates collected showed a low genetic diversity and important persistence characteristics. *L. monocytogenes* isolates were not stochastically distributed on the surfaces: the isolates were detected on three out of six production lines, each associated with a specific meat cut: the half carcasses, the bostons and the picnics. MaAsLin biomarker analysis identified the taxa *Veillonella* (*p* ≤ 0.0397) as a bacterial determinant of the presence of *L. monocytogenes* on processing surfaces. The results of this study revealed a heterogenous contamination pattern of the processing surfaces by *L. monocytogenes* and targeted a bacterial indicator of the presence of the pathogen. These results could lead to a better risk assessment of the contamination of meat products.

## 1. Introduction

*Listeria monocytogenes* is a foodborne pathogen and the etiological agent of human listeriosis. In its invasive form, this disease affects immunocompromised individuals, the elderly population, pregnant women, and newborns. Within this population *Listeria monocytogenes* can cause septicemia, meningoencephalitis and miscarriage, and presents a high mortality rate (15–20%) [1,2]. The main route of human contamination is through the consumption of food contaminated by the pathogen.

Multiple listeriosis outbreaks associated with Ready-To-Eat meat (RTE) products have been reported across the globe [3,4]. In 2008, Canada experienced the largest outbreak in its history. It was linked to the contamination of deli-meat by the food processing environment (57 cases, 24 deaths) [5,6,7]. The post-thermal treatment contamination of the meat products during processing procedures is frequently involved as the cause of outbreaks. For such cross-contamination to occur it is essential that *L. monocytogenes* has been introduced into the facility, leading to the colonization of the RTE processing environment [8]. Nastasijevic et al. (2017) reported two pairs of *Listeria monocytogenes* isolates, genetically identical, harvested from a dispatch unit of a RTE facility and from a water drain and a floor–wall junction of a slaughterhouse [8]. Bolocan et al. (2015) showed that a new meat processing facility can be colonized by *L. monocytogenes* as soon as contaminated raw materials are brought into the production environment [9]. Thus, the tracking of the spreading routes of *L. monocytogenes* at the slaughterhouse is crucial to prevent the introduction of the pathogen to the RTE processing environment and its transfer to RTE meat products [8].

It has been shown that clones of *Listeria monocytogenes* harvested from the food processing environment possess differences in their pathogenic potential or survival rate. Isolates from the serotypes 4b, 1/2a and 1/2b are overrepresented in human listeriosis. These three serotypes are responsible for more than 95% of human listeriosis cases [10,11]. 

Different rapid first line molecular and classical typing methods such as PCR-serogrouping can be used for the screening of multiple isolates and to give a general idea of the diversity of the *L. monocytogenes* population present in an environment [12]. Moreover, the recent implementation of the *Listeria monocytogenes* Core Genome Multilocus Sequence Typing (cgMLST) allows for a more powerful detection of clusters of listeriosis as well as for the identification of multiple genes of interest [13]. The Listeria Pathogenicity Island-I (LIPI-1) is an important virulence marker which includes, among others, the gene coding for the internalin A. LIPI-1 is regulated by the *prfA* gene which signals the transition between the extracellular and the intracellular lifestyles of the bacteria [14]. It is known that the gene encoding internalin A can harbor premature STOP codons (PMSC) that can lead to a decrease of the virulence of the bacteria [15,16,17,18]. Two Survival Islets, Stress Survival Islet 1 (SSI-1) and Stress Survival Islet 2 (SSI-2), are known to be important in the survival of *Listeria monocytogenes* to stresses encountered in the production environment [19]. The *bcrABC* genes have also been identified to be beneficial to the bacteria regarding its tolerance to benzalkonium chloride [20,21,22]. Taken together, the results of first lines typing methods, the identification of virulence, antimicrobial resistance, and stress tolerance genes by cgMLST, as well as the assignment of cgMLST types, could provide a prediction of the diversity of the *L. monocytogenes* population in a given environment, and thus clues as to the involvement of persistence or recurrence scenarios. 

However, the results of several studies suggested that no individual characteristic responsible for strain-to-strain variation is sufficient to explain why some *Listeria monocytogenes* subtypes can better survive in the food processing environment [23,24]. The resident microbiota has been put forward as a hypothesis to explain the presence of *L. monocytogenes* in the processing environment. Indeed, the background microbiota is known to play a role in the protection of pathogens. [25]. In food facilities, *Listeria monocytogenes* can be found associated with other microorganisms in multispecies biofilms [26,27]. The interactions between the microorganisms and *Listeria monocytogenes* have been shown to modify the capacity of the pathogen to colonize facilities [28,29,30,31], implicating positive effects such as resistance to disinfectants and enhanced adherence to surfaces [32] as well as negative effects such as nutrient-limiting conditions and the production of antilisterial compounds [33,34]. 

Several studies have shown that during the processing of carcasses at the slaughterhouse, a microbiota can survive, come into contact with food-contact surfaces such as conveyor belts, detach and therefore contaminate the food product [11]. In fact, in the case of *Listeria monocytogenes*, the most frequent pathway of contamination of food products is by cross-contamination with food processing surfaces [29,35,36]. This type of transfer of the pathogen has been identified as the cause of outbreaks of listeriosis [37]. Biofilms can establish themselves in irregularities on the surface of conveyors and thus become sites of contamination during the passage of food products [38,39,40]

In a recent review on the microbial diversity and ecology of biofilms associated with *L. monocytogenes* in the industry environment, Fagerlund et al. (2021) highlighted the need for high throughput sequencing (HTS) technology approaches for the detection of interactions between the members of microbial communities present in the biofilms found on industrial surfaces. [41]. Several studies have carried out the analysis of the microbiota and the detection of *Listeria monocytogenes* for the same surface or sample [42,43,44]. However, most of them have chosen classical culture-dependent techniques for the characterization of the microbiota. Thus, the results obtained from these studies represent an approximation of the composition of the microbiota since only a small portion of cultivable bacteria are taken into account [45]. 

Advances in HTS sequencing have resulted in the development of rapid and efficient methods for the characterization of the composition of microbial communities. These HTS approaches allow the detection of non-cultivable bacteria as well as the detection, due to their high sensitivity, of nondominant bacteria. In recent years, few studies have employed HTS technology to attempt the identification of the bacterial genus potentially implicated in the presence of *Listeria monocytogenes* on surfaces in the food industry [28,39,45,46,47,48]. These studies successfully identified dominating bacteria found in the environment microbiota when *Listeria monocytogenes* was present or absent. Rodriguez-Lopez et al. (2019) identified the *Actinobacteria* as the most present taxa in a sample from a meat facility surface [45]. Liu et al. (2016) showed that *Pseudomonas psychrophila*, *Pseudomonas* sp., *Klebsiella* sp., *Klebsiella oxytoca* and *Aeromonas hydrophila* were dominant in the microbial community of *Listeria*-positive drain samples [46]. Another study from Tan et al. (2019) revealed a distinct microbiota in a facility with a higher occurrence of *Listeria monocytogenes* [28]. This distinct microbiota was predominated with Pseudomonadaceae and the fungal family Dipodascaceae. These results reinforce the idea that the composition of the environmental microbiota may be of interest for the identification of contamination by *L. monocytogenes* [41]. 

However, all the above studies focused on dominant members of the microbiota community found at the same location as *L. monocytogenes*, while nondominant members may play an important role in the establishment of the pathogen [41]. In addition, these studies rely on a correlation between the simultaneous presence of *L. monocytogenes* and certain dominant members of the accompanying microbiota, without ensuring that the observed changes are not due to confounding factors such as time and location. Knowing that there is evidence of microbial niche partitioning in the food processing environment ([49,50] and since the bacteria identified as associated with the presence of *Listeria monocytogenes* have been, until now, those which usually dominate in the production environment, there is a need for studies that can identify bacterial determinants positively and negatively associated with the presence of the pathogen on a same surface, in a restricted area [51] and in the same time frame [52]. 

The objectives of this study were to (i) characterize the genomic diversity of *Listeria monocytogenes* isolates harvested from the six conveyor belts of the cutting room of a swine slaughterhouse, (ii) to evaluate the heterogeneity of the spatial and temporal contamination of these surfaces by the pathogen and (iii) to identify, using 16S rRNA amplicon sequencing, microbial determinants of the presence or absence of the bacteria. We think that the outcomes of this study will allow a more accurate understanding of the contamination of the pork raw material by *L. monocytogenes* and ultimately contribute to the improvement of the management of food safety regarding the pathogen. 

## 2. Results

### 2.1. Listeria monocytogenes Detection

**Figure 1:**
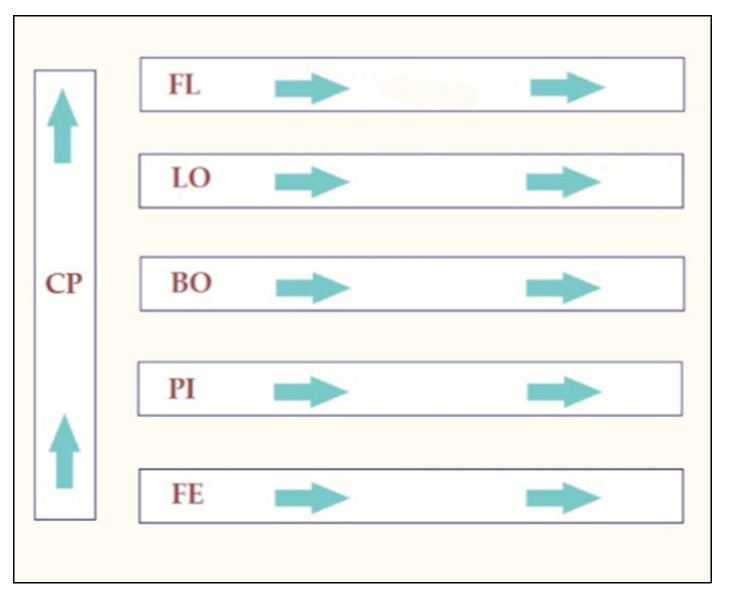
Organization of the conveyor belts in the cutting room of the swine slaughterhouse. A specific cut of meat circulates on each conveyor. CP: half carcass, FL: belly, LO: loin, BO: boston, PI: picnic, FE: ham. The arrows indicate the direction of the flow of the meat products.

**Figure 2:**
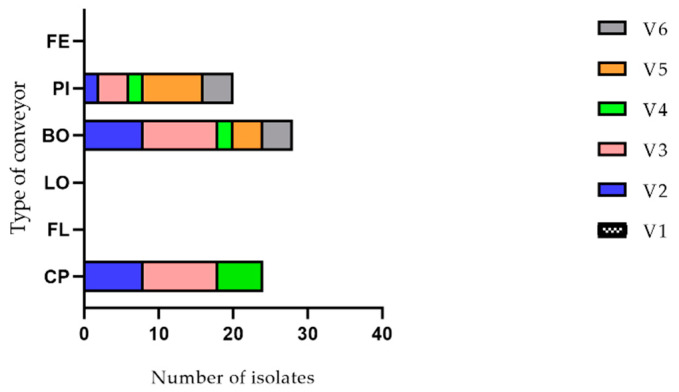
Number of *Listeria monocytogenes* isolates collected per conveyor and per visit. The different conveyors are shown on the ordinate axis. Each visit is represented by a color according to the legend.

A total of 72 *Listeria monocytogenes* isolates were collected from the conveyor belt surfaces of the cutting room of a swine slaughterhouse during six visits (see Figure 1). The isolates were only found on three out of six conveyors: 24 on the main conveyor (half-carcasses), 28 on the conveyor for bostons and 20 on the conveyor for picnics. The conveyor for bellies, the conveyor for loins and the conveyor for hams were systematically negative. Eighteen isolates were found during the second visit, and 24, 10, 12 and eight isolates were found during the third, the fourth, the fifth and the sixth visits, respectively (see Figure 2). The number of isolates per conveyor was shown to be significantly different (*p* = 0.003) while the difference among the number of isolates per visit did not show a significant difference (*p* = 0.2105) using ANOVA with a significant level of 0.05 and the Geisser–Greenhouse correction. 

### 2.2. Listeria monocytogenes Classical and Molecular Characterization

#### 2.2.1. *Listeria monocytogenes* PCR-Serogroups

Molecular and classical serotyping identified five different serotypes: 1/2a, 1/2c, 3a, 3c and 4b (see Table S1). Serotype 1/2a was the most frequently isolated serotype and represented 61.1% (n = 44) of the isolates. Serotypes 1/2c, 3a, 3c and 4b, respectively, represented 18.1% (n = 13), 11.1% (n = 8), 5.6% (n = 4) and 4.2% (n = 3) of the isolates. Serotypes 1/2a, 1/2c and 3a (lineage II) isolates were found on all the positive conveyors while serotype 3c (lineage II) isolates were only found on the main conveyor and the conveyor for the bostons. Serotype 1/2b (lineage I) isolates were only detected on the conveyor for the picnics. 

#### 2.2.2. InlA Sequencing

The *inlA* gene of each isolate was sequenced with the Sanger method. Most of the isolates, 86.1% (n = 62), harbored a premature STOP codon. All the serotype 3c isolates (n = 4) presented a complete *inlA* as well as two serotype 3a isolates (n = 8), three serotype 1/2a isolates (n = 44) and one serotype 1/2b isolate (n = 3) (See Table S1). All the PMSC were of type 3 (699AA) except for one isolate that harbored a type 10 (676AA) PMSC type. 

#### 2.2.3. Determination of the Ability to Form Biofilm

**Figure 3:**
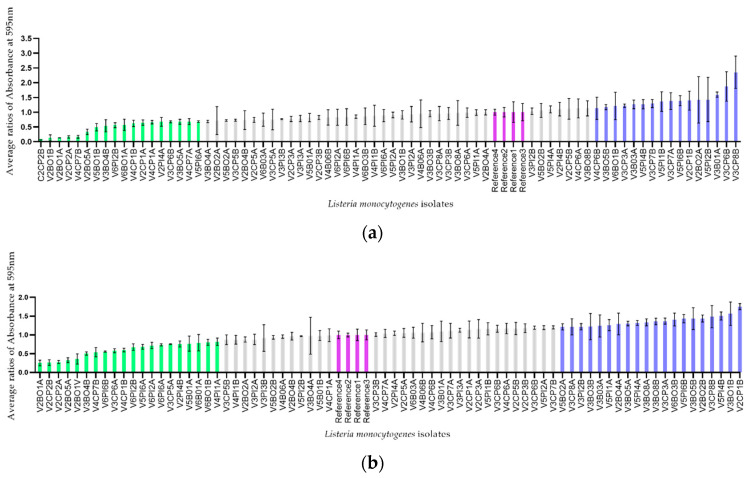
(**a**) Characterization of the ability of the isolates to produce a biofilm after seven days of incubation at 12 °C; (**b**) Characterization of the ability of the isolates to produce a biofilm after two days of incubation at 30 °C. The isolates classified as low biofilm producers are represented in green, the moderate biofilm producers in gray, the high biofilms producers in blue and the reference strain from each microtiter plate in purple.

The ability of each isolate to produce a biofilm at 30 °C and 12 °C (in order to get closer to the temperature found in a cutting room at the slaughterhouse) on a microtiter plate was evaluated. Crystal violet assays were performed and the measurements of the absorbance at 595 nm of the level of coloration resulting from the dissolution of the colored biofilm by the addition of alcohol were obtained. To control the possible variation in the results caused by the use of multiple microtiter plates, the level of biofilm production of each isolate was expressed as a proportion. This proportion had as numerator the absorbance of each isolate and had as denominator the absorbance of a reference strain (C.R.S.V. 3C15). The isolates were distributed according to their distance from the reference strain. The quarter of isolates with the lowest ratios were classified as low biofilm producers, the quarter of isolates with the highest ratios were classified as high biofilm producers and the isolates in the middle half were classified as moderate biofilm producers (see Figure 3). The average ratios obtained at 30 °C ranged from 0.25 to 1.56 and the average ratios obtained at 12 °C ranged from 0.03 to 1.87. 

#### 2.2.4. MLST and cgMLST Characterization, Virulence, Antimicrobial Resistance and Stress-Related Genes

**Figure 4:**
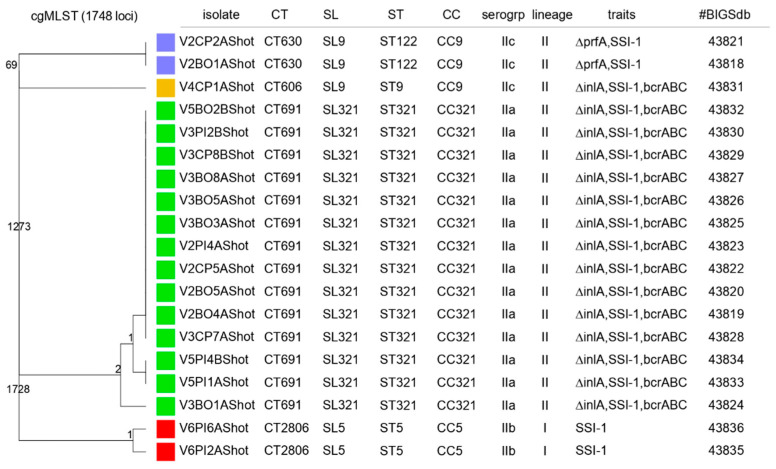
Dendrogram representing clustering of *L. monocytogenes* isolates based on the cgMLST profiles. The different cgMLST profiles are represented by a specific color. Column: 1—Isolates ID, 2—cgMLST types, 3—Sublineages, 4—Sequence types, 5—Clonal complexes, 6—Serogroups, 7—Traits, 8—Pasteur Institute BIGSdb-*Lm* ID. In the nodes, the numbers of allelic differences are indicated.

Nineteen isolates were selected for characterization by MLST and cgMLST. Those isolates were selected to represent the diversity of the different strains of *L. monocytogenes* found in the cutting room of the slaughterhouse. Four allelic profiles (STs) were identified: ST122 (10.5%, n = 2), ST9 (5.3%, n = 1), ST321 (73.7%, n = 14) and ST5 (10.5%, n = 2). *Listeria monocytogenes* isolates identified by PCR-serogrouping as serotypes 1/2a, 1/2c and 3a were classified to ST321 sequence type, and the isolates belonging to serotypes 3c and 1/2b were classified to ST122 and to ST5 sequence types, respectively. The different isolates were grouped into three clonal complexes: CC9, CC321 and CC5 (see Figure 4). *Listeria monocytogenes* isolates were classified into four CTs (CT630, CT606, CT691 and CT2806) and into three SLs: SL9 (15.8%, n = 3), SL321 (73.7%, n = 14), SL5 (10.5%, n = 2). 

Seventy-eight point nine percent (n = 14) of the isolates harbored a PMSC in the *inlA* which is consistent with the Sanger sequencing results apart from the V6PI2A isolate whose *inlA* was characterized as complete by sanger sequencing and truncated by WGS. The two isolates from the L1-SL9-ST122-CT630 harbored a deletion of the transcriptional activator PrfA. The efflux pump system (*bcrABC*) known to confer benzalkonium chloride tolerance was detected in 78.9% (n = 15) of the isolates. More precisely, the *bcrABC* system was identified in one isolate from the L1-SL9-ST9-CT606 and the 14 isolates from the L1-SL321-ST321-CT691. The stress survival islet 1 (SSI-1) was detected in all 19 isolates.

### 2.3. Microbiota Analysis

#### 2.3.1. Sequencing Data

A total of 11,180,771 sequences were obtained from the sequencing. After the cleaning, this number was reduced to 7,198,363 sequences with an average of 23,437 sequences per sample grouped into 10,280 OTUs. The lowest and the highest number of sequences found in a sample were, respectively, 10,087 and 41,555. The experimentation controls showed an average of 16,536 sequences, the sequencing controls an average of 7692 sequences and the ZymoBIOMICS Microbial Community DNA Standard positive controls an average of 17,733 sequences. The controls were satisfactory including the positive controls in which the eight bacterial genera composing the mock community were found after sequencing in expected proportions. For the remainder of the analysis, sequences from the controls were removed. 

#### 2.3.2. Alpha Diversity

For alpha-diversity analysis, a subsampling was conducted, and the diversity indices were calculated with 1000 iterations based on the lowest sequence number per sample (10,087 sequences). Three alpha-diversity indices were used: the average number of observed OTUs (Observed), the evenness of the OTUs found in the samples (Shannon evenness) and the diversity of these OTUs (inverted Simpson’s index). Measurements of alpha-diversity indices of the *Listeria monocytogenes* positive samples and the *Listeria monocytogenes* negative samples were compared using Student’s t-test with a significance level of 0.05 (see Figure S1 and Table 1). Student’s t-tests revealed no difference in alpha diversity with the Observed index (*p* = 0.359), with the Shannon evenness (*p* = 0.195) and with the Inverted Simpson’ index (*p* = 0.213).

#### 2.3.3. Beta Diversity

**Figure 5:**
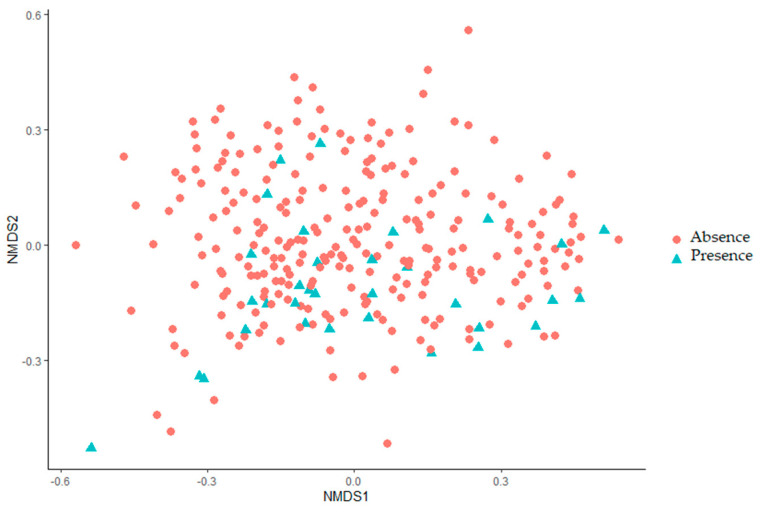
Non-Metric Multidimensional Scaling Graph (NMDS) of the microbiota structures of the *Listeria monocytogenes* positive samples and the *Listeria monocytogenes* negative samples using the Bray–Curtis index. Each point represents a sample, and each condition is represented by the combination of a symbol and a color. Blue triangles: negative *Listeria monocytogenes* microbiota in samples, red circles: positive *Listeria monocytogenes* microbiota in samples.

Beta-diversities were compared between the *Listeria monocytogenes* positive samples and the *Listeria monocytogenes* negative samples, after a subsampling and the diversity indices were calculated with 1000 iterations based on 10,087 sequences. The similarity of the microbiota structures for the two conditions at the OTU level was compared with an ANOVA using the Jaccard index based on the presence/absence of OTUs, and the Bray–Curtis index based on the relative abundance of OTUs and visualized with 2D nonmetric multidimensional scaling graphs (NMDS) (see Figure 5 and Figure S2). No statistically significant difference was found between the *Listeria monocytogenes* positive samples and the *Listeria monocytogenes* negative samples within a same visit (*p* = 0.08192) or the same conveyor (*p* = 0.05095) although the *p*-value of the latter condition was very close to the statistically significant threshold.

**Figure 6:**
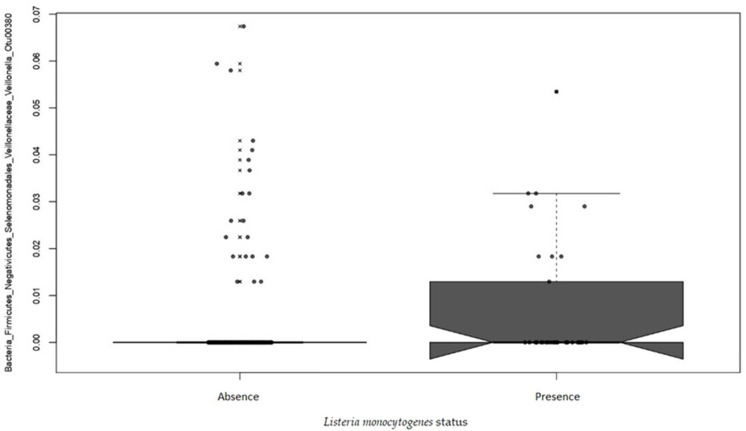
Multivariate association with linear model analysis (MaAsLin) graph of the association between the presence of *Listeria monocytogenes* and the *Veillonella* taxa OTU00380. The boxplot summarizes the distribution of the data. Outliers are represented by x. The jittered raw data are represented by dots to highlight the noise and the number of observations.

Multivariate association with linear model analysis (MaAsLin) was conducted to identify OTUs that were significantly associated with the presence or the absence of *Listeria monocytogenes* within the same visit and the same conveyor. The OTU 00380 associated with the bacterial genera *Veillonella* showed a positive association with the presence of *Listeria monocytogenes* (*p =* 0.0397) (see Figure 6).

## 3. Discussion

In our study, a total of 72 isolates collected from 36 *Listeria monocytogenes* positive samples were harvested from food-contact conveyor belt surfaces corresponding to an incidence of 12.24% (36 out of 294 samples). This incidence rate is relatively low compared to the incidence of *Listeria monocytogenes* reported in other studies carried out at the slaughterhouse or at processing meat plants. Sala et al. (2016) found an incidence of 25.8% in environmental samples of a swine slaughterhouse [53]. An incidence of 33.3% was associated with the conveyor belt surfaces. Autio et al. (2000) conducted a survey in ten pig slaughterhouses that revealed an overall incidence of 16.77% of *L. monocytogenes* (5 out of 73 samples). The positive samples sites included saws, drains, doors and tables [54]. Muhterem-Uyar et al. (2015) reported an incidence of *Listeria monocytogenes* of 18.8%. 26.5% and 50.5% in the environment of three meat processing plants, respectively. The highest incidence rate was associated with the fact that the slaughter and the processing were performed together at that facility [55]. Bolocan et al. (2015) found an incidence of 22.9% on food-contact surfaces including conveyors, tables, slicers, grinders and knives in a meat plant producing ready-to-eat food as well as food requiring cooking while Rodriguez-Lopez et al. (2019) reported an incidence of 36.3% in environmental samples coming from the surfaces of meat processing industries [45,56]. 

The origin of the sampling sites can be put forward as a hypothesis to explain the low incidence obtained in our study. Our sampling took place in a cutting room, an environment in contact with meat products but with a very little exposure to viscera and other animal wastes. In addition, it has been reported that the degree of contact of a surface with the food products is not predictive of the level of contamination by *Listeria monocytogenes* [53]. In fact, some studies showed a higher recovery rate of the pathogen on non-food contact surfaces than on food-contact surfaces [53]. However, some studies assert that contact with raw material must be involved in the contamination by *Listeria monocytogenes* [57]. The variations in the sampling methods of the different studies as well as country-specific washing and disinfection measures may have also contributed to differences in observed incidences.

The *Listeria monocytogenes* isolates in our study were all found on the same three conveyors: the main conveyor (CP), the conveyor for the bostons (BO) and the conveyor for the picnics (PI). The conveyor for bellies (FL), the conveyor for loins (LO) and the conveyor for hams (FE) were systematically negative to *L. monocytogenes* even though the same number of samples were taken on all conveyors (48) except for the main conveyor (54). To our knowledge, this is the first time that a study reports a clear preferential localization of *Listeria monocytogenes* associated with surfaces with identical physical characteristics. In fact, the sampled conveyors all presented the same design and could only be differentiated by the type of meat cut circulating on each of them. Evidence of differences among the microbiota of conveyor belts, harvested on blood agar, has been reported by Fagerlund et al. (2017) [39]. One conveyor associated with a recurrent presence of *Listeria monocytogenes* presented a very diverse microbiota dominated by *Mycobacterium* and *Epilithonimonas*. De Filippis et al. (2013) showed an association among the microbiota found on pieces of meat from the same cut despite belonging to different beef carcasses [58]. Thus, the cuts of the carcass seem to affect the contaminating microbiota found on beef meat. Together these results suggest a role of the background microbiota associated with the different meat cuts in the sheltering of *Listeria monocytogenes* on certain preferential sites. 

The impact of the orientation of the carcass hanging upside down as it enters the cutting room could also represent a hypothesis for the presence of *Listeria monocytogenes* on conveyors associated with certain pieces of meat. Indeed, the pathogen could be brought to run together with other microorganisms towards the bottom of the carcass, thus preferentially contaminating the parts associated with the top of the animal. Applied to the context of our study, this hypothesis is consistent with the contamination of the conveyors for bostons and picnics but does not explain why conveyors associated with center pieces such as bellies and loins were not found as contaminated. 

Another hypothesis that can be considered in the contamination by *L. monocytogenes* of specific conveyors is the presence of the skin on the pieces of meat that circulate on their surfaces. The half carcasse, the boston, the picnic and the ham are usually pieces of meat with skin, while the bellies and the loins typically do not include skin. Again, applied to the context of the study, this hypothesis is consistent with the contamination of the main conveyor, the conveyor for the bostons and the conveyor for the picnics, but does not explain why the conveyor for the hams was not identified as contaminated by the pathogen. 

In our study, the serotype 1/2a was the most dominant representing 61.1% of isolates. Together, serotypes belonging to lineage II (1/2a, 1/2c, 3a and 3c) account for 95.8% of the isolates collected. Four-point two percent of the isolates were associated with serotype 4b (lineage I). These results are not surprising since it is known that lineage II strains are widespread in the natural environment, on farms, in the production environment and are often associated with sporadic cases of listeriosis while lineage I is overrepresented in human listeriosis cases and outbreaks [59]. Martin et al. (2014) also found the isolates belonging to serotypes 1/2a and 1/2c to be dominant in the meat processing environment counting for 36.8% and 34% of the isolates, respectively. The authors found a low percentage of isolates of serotype 4b (11.3%) and 1/2b (17.9%) but no 3c isolate [11]. In another study, Nastasikevic et al. (2017) reported that the eight *Listeria monocytogenes* isolates harvested from the meat processing environment were associated with serotypes 1/2a, 1/2c and 4b [8]. Serotype 3c has also been reported to be meat-associated, although recovered with a low frequency [60]. 

In our study, most of the isolates, 86.1% (n = 62), harbored a PMSC in their *inlA* gene sequence. Interestingly, the production of a complete internalin A was mostly found in serotypes less frequently associated with the production environment: in all the serotype 3c isolates (n = 4), two serotype 3a isolates (n = 8), three serotype 1/2a isolates (n = 44) and one serotype 1/2b isolate (n = 3). Nightingale et al. (2020) reported that the PMSCs in the *inlA* gene represented a virulence-attenuated subpopulation of *Listeria monocytogenes* strains, commonly associated with food. The authors also suggested the high occurrence of several distinct PMSC mutation points could be the result of a positive selection for the loss of the cell-wall-anchored InlA in some environments [17]. 

The ability of the 72 isolates to produce a biofilm at 30 °C and 12 °C was evaluated. Interestingly, the level of biofilm production by low and medium producers was generally higher at 30 °C, but the level of biofilm production by strong producers was higher at 12 °C. This observation could be explained by the fact that the isolates come from a cutting room and several strains are therefore more adapted to low temperatures [61]. However, the incubation time may have also had an impact on the biofilm density. 

Nineteen isolates among the 72 characterized were selected to be sequenced and analyzed by cgMLST. A very low genomic diversity was revealed by the analyses. This low diversity can be explained by a persistence of the isolates in the production environment. Indeed, the knowledge that the isolates were collected over a long period of time, in the same cutting room, as well as of their important persistence characteristics and their attribution to old clonal complexes allows us to set the hypothesis of a persistence scenario rather than a recurrent introduction [35]. 

The analysis classified the isolates in three clonal complexes: CC9, CC321 and CC5. The clonal complex CC5 was associated with the isolates belonging to the IIb PCR-serogroup. These isolates were linked to the sublineage SL5. Studies have reported that the CC5-SL5 isolates show a better survival rate in the food processing environment than other isolates [12]. Unlike the study conducted by Muhterem-Uyar et al. (2018), our isolates belonging to the SL5 sublineage did not harbor the *bcrABC* cassette responsible for tolerance to benzalkonium chloride [62]. The CC5 clonal complex was shown to be associated with the epidemic clone ECVI which was linked to listeriosis outbreaks [62]. Three of the isolates of our study were associated with the clonal complex CC9 and the sublineage SL9. Those isolates were linked to the IIc PCR-serogroup. The clonal complex CC9 has been characterized as hypovirulent and is part of the expansion of some lineage II CCs associated with an adaptation to the food environment [62,63]. The CC321 clonal complex has also been reported to be highly prevalent in the food-associated isolates [64]. In accordance with what has been reported in the literature, our isolates associated with the C321 clonal complex were predominantly of serotype 1/2a [64].

The isolates showed characteristics of attenuated virulence. Fourteen isolates out of nineteen harbored PMSCs in their *inlA* gene. The Sanger sequencing and the characterization by cgMLST showed the same results with regard to the length of the internalin A apart from the V6PI2A isolate whose InlA was characterized as complete by sanger sequencing and truncated by cgMLST. A mutation following a series of freezing and thawing through time could have occurred, although a maximum of three thawing per aliquot were performed and a collection of colonies from a single plate was used for the sequencing. An analysis error could also have been involved. 

The two isolates from the L1-SL9-SL122-CT630 harbored a deletion in the sequence of the transcriptional activator PrfA, the most important regulator of *L. monocytogenes* virulence and are therefore avirulent [65,66]. The efflux pump system (*bcrABC*) known to confer benzalkonium chloride tolerance was detected in 78.9% (n = 15) of the isolates making these isolates more likely to survive washing and disinfection procedures [20]. The stress survival islet 1 (SSI-1) was detected in all of the 19 isolates. It has been shown that SSI-1 may contribute to the survival of *Listeria monocytogenes* under suboptimal conditions as found in the food processing environment [67]. 

The characteristics harbored by the isolates should allow them to be found on any of the six conveyors sampled, whereas they were systematically found on only three of these conveyors. The accompanying microbiota hypothesis was therefore explored as a possible cause for the heterogeneity in the localization of the pathogen. 

In our study, no statistically significant difference was found between the *Listeria monocytogenes* positive samples and the *Listeria monocytogenes* negative samples for the alpha diversity. These results suggest that the number of different bacterial genera and the uniformity in the number of representatives of each of these bacterial genera are not affected by the presence of the pathogen. In addition, no statistically significant difference was found between the *Listeria monocytogenes* positive samples and the *Listeria monocytogenes* negative samples for the beta diversity, indicating that the structure of the majority and the minority bacterial populations are not affected by the presence of *Listeria monocytogenes*.

These results must be interpreted in the context of this study. Indeed, samples very close to each other belonging to the same area and collected at the same time were compared in our study. It can be hypothesized that differences in the microbiota structure of such similar samples may be too minor to be revealed by alpha and beta diversity analyses. Although several studies have reported changes in the identity of the dominant species in *Listeria monocytogenes* positive samples, none of these studies, to our knowledge, have considered the impact of confounding factors such as time and sample location as performed in our study [28,39,45,46,47,48].

An association in terms of relative abundance was found using the Multivariate association with linear model analysis (MaAsLin). The OTU00380 representing a *Veillonella* taxa was associated with the presence of *Listeria monocytogenes* independently of the visit or the conveyor. The higher abundance in *Listeria monocytogenes* positive samples of this non-dominant OTU in the total microbiota of the conveyor surfaces may not have been sufficient on its own to lead to a discernible change in the microbiota structure. *Veillonella* spp. are strictly anaerobic Gram-negative cocci often found in the microbiota of the mouth and gastrointestinal tract of humans and animals, including the pig. A study by Crespo-Piazvelo et al. (2018) showed the presence of *Veillonella* spp. in the ileum of pigs. The presence of the bacteria was correlated with the presence of *Actinobacillus* [68]. Another study by Huang et al. (2019) revealed an increase of *Veillonella* spp. in diarrheal pigs infected by Porcine epidemic diarrhea virus (PEDV) [69]. Regarding foodborne pathogens, studies performed by Hinton JR. et al. (1993, 1995), reported that the production of acetate and propionate by *Veillonella* was correlated with an inhibition of the growth of *Salmonella* Typhimurium, *Salmonella* Enteritidis, *Escherichia coli* O157:H7 and *Pseudomonas aeruginosa* [70,71,72]. The use of anaerobic bacteria such as *Veillonella* as components of probiotic cultures has been suggested in order to reduce the colonization of young chicks by *Salmonella* [70]. We believe that the results of our study that successfully identified a positive correlation between the presence of *Veillonella* and *Listeria monocytogenes* demonstrate the importance of evaluating the impact of such probiotics on a wide range of food pathogens. To our knowledge, no correlation has been reported to date between the presence of *Listeria monocytogenes* and *Veillonella* spp. Our study is the first one to identify the *Veillonella* genera as a possible indicator of the contamination of food processing surfaces by *Listeria monocytogenes*.

## 4. Materials and Methods

### 4.1. Sampling

Six visits (distributed over a period of six months) were made to the cutting room of a swine slaughterhouse. A total of 300 conveyor belt sample surfaces (900 cm^2^ per sample) in contact with the meat products were collected firstly by brushing the conveyor belts surfaces and secondly by rubbing the surfaces with wipes (Innovation Diagnostics, Saint-Eustache, QC, Canada). Ten mL of a neutralizing solution was previously added to the wipes to avoid the potential effect of cleaner and disinfectant residues on the bacteria (Innovation Diagnostics, Saint-Eustache, QC, Canada). The samples were distributed among the six conveyor belts of the cutting room. Different meat cuts circulate on each conveyor. In total, 54 surface samples were taken on the main conveyor (CP), 48 on the conveyor for bellies (FL), 48 on the conveyor for loins (LO), 48 on the conveyor for the bostons (BO), 48 on the conveyor for picnics (PI) and 48 on the conveyor for hams (FE) (see Figure 1). One experimental control per visit was also taken. The experimental controls were wipes and brushes taken out in the cutting room but that were not in contact with the conveyor belts. All the samples were transported at 4 °C and processed within three hours in the laboratory. 

### 4.2. Listeria monocytogenes Detection

At the arrival at the laboratory, each wipe was cut in half under sterile conditions. The first half was used for the detection of *Listeria monocytogenes* and the second half was used for the harvesting of the total microbiota. One half of each wipe was added to 100 mL of UVM 1 modified broth (Biokar diagnostics, Allonne, France) and incubated at 30 °C for 48 h. A second enrichment was performed in Fraser broth (Biokar diagnostics, Allonne, France). For this purpose, 100 µL of inoculated UVM 1 of each sample was added to 10 mL of Fraser broth and incubated for 24 h at 30 °C. One hundred µL of each inoculated Fraser broth was put on the selective chromogenic medium RAPID’ *L. mono* (BioRad Laboratories inc., Montreal, QC, Canada) and incubated for 24 h at 37 °C. For each positive sample, two presumptive colonies were plated on blood agar and incubated for 24 h at 37 °C. Two colonies per blood agar plate were then tested for rhamnose fermentation by adding each colony to 5 mL of Purple broth base (HiMedia Laboratories, Mumbai, India). The *Listeria monocytogenes* identity as well as the serogrouping of the isolates were confirmed by PCR as described below. The confirmed *L. monocytogenes* were stored at −80 °C for further analysis.

### 4.3. Total Microbiota Harvesting

The second half of the wipes was added to 25 mL of a DNA preservation solution made of Tris-HCL [10 mM], EDTA [10 mM] and NaCl [0.85%]. Each sample (half wipes in 25 mL of the DNA preservation solution) was stomached (Biomérieux Canada, QC, Canada) for one minute to dislodge the microorganisms present on the half wipe and to homogenize the solution. Twenty mL of the homogenized solution was transferred into two falcons of 15 mL (Sarstedt Inc., Saint-Laurent, QC, Canada) at the rate of 10 mL per falcon. The falcons were then centrifuged at 5000 rpm for 20 min at 4 °Celsius (VWR, Saint-Laurent, QC, Canada). The bacterial pellets obtained were individually stored at −80 °C until DNA extraction and purification.

### 4.4. Listeria monocytogenes Classical and Molecular Characterization

The isolates were cultured on blood agar at 37 °C for 24 h and a loopful of bacteria was transferred into 50 µL of a 6% chelex solution. The inoculation solution was vortexed (Fisher Scientific, Saint-Laurent, QC, Canada) for 10 s followed by two dry baths: 30 min at 55 °C and 15 min at 98 °C, respectively. The solution was then centrifuged during 5 min at 14,000 rpm and maintained at 4 °C. The supernatant was collected and conserved at −80 °C for further analysis. 

The presence of DNA was validated by gel electrophoresis (3% of agarose). *L. monocytogenes* isolates were typed by PCR-serogroups using the molecular serotyping scheme as previously described by Kérouanton et al. (2009) [73]. In order to distinguish the serovar 1/2a from 3a, 1/2c from 3c, 1/2b from 3b and 7, or 4b from 4d and 4e agglutination against discriminatory O serum (OI, OVII, OVIII; Oxoid Thermo Fisher Scientific, Nepean, ON, Canada) was conducted as previously described by Burall and al. (2011) [74]. 

The *inlA* gene of each isolate was sequenced using the Sanger method at the Centre d’Innovation Génome Québec (Applied Biosystems 3730xl DNA analyzer) using four overlapping amplifications. The sequences were aligned and screened for premature STOP codon using Sequencher 5.4.6 software with the sequence of *inlA* of *L. monocytogenes* EGD-e (NCBI: NC_003210.1) used as a reference. 

The ability of each isolate to produce a single species biofilm at 30 °C and 12 °C on a microtiter plate was evaluated. The isolates were cultured on blood agar at 37 °C for 24 h. Subsequently three colonies per isolate were used to inoculate 10 mL of 6% TSBYE broth (Becton Dickinson Company, Mississauga, ON, Canada). After 24 h at 37 °C the absorbance at 600 nm was calculated. One hundred µL of the TSBYE broths was then put in 10 mL of BHI (Becton Dickinson Company, Mississauga, ON, Canada) and incubated for 24 h at 37 °C. Afterwards, 100 µL of the BHI broths was used to inoculate three consecutive wells of two plates. One plate was incubated at 30 °C for 48 h and the second plate was incubated at 12 °C for one week. The plates were incubated under humid conditions. Crystal violet (1%, filtered at 0.45 µM) assays were performed. Briefly, the medium was removed and three washes with 150 uL of sterile water were then performed. After each wash, the wells were emptied. A drying time of 10 min at room temperature was then observed. Next, 50 µL of crystal violet was added to each well and a waiting time of 30 min at room temperature was carried out. Three washes with 150 µL of sterile water were again performed and the wells were emptied after each wash. A drying time of 10 min at room temperature was again observed. Finally, 200 µL of 90% ethanol was added to each well 30 min before the reading of the absorbance at 595 nm (Power Wave X 340, Bio-Tek Instruments, INC). Each isolate was included in triplicate in the microtiter plates. Absorbance measurements were corrected by the blank which consisted of a well without biofilm that underwent crystal violet staining. The average of the calculated optical density was used as result. During the waiting times the microtiter plates were protected from light. To control the possible variation in the results caused by the use of multiple microtiter plates, the level of biofilm production of each isolate was expressed as a proportion. This proportion had as numerator the absorbance of each isolate and had as denominator the absorbance of a reference strain (C.R.S.V. 3C15). The reference strain was included in each microtiter plate, thereby allowing the expression of the absorbance of each isolate from a microtiter plate over the absorbance of the reference strain included in the same microtiter plate. The reference strain was a *Listeria monocytogenes* strain isolated in a previous study and that has been characterized as a moderate biofilm producer. The isolates were distributed according to their distance from the reference strain result. The quarter of isolates with the lowest ratios were classified as low biofilm producers, the quarter of isolates with the highest ratios were classified as high biofilm producers and the isolates in the middle half were classified as moderate biofilm producers. 

### 4.5. Selection, DNA Isolation, Library Preparation and Sequencing of the L. monocytogenes Isolates

Nineteen isolates were selected for characterization by cgMLST. These isolates taken together represented all the serotypes identified in the context of this study, the different forms of InlA (completed, truncated) found and all the categories of production of biofilm at 12 °C and 30 °C (weak, moderate, high) identified. Isolates from each conveyor positive to *Listeria monocytogenes* as well as from each positive visit were included within these 19 isolates. DNA extraction was performed using the MasterPureTM DNA Purification kit (Épicentre, BC, Canada) according to the instructions of the manufacturer instructions. The Ready-LyseTM Lysozyme was used in a prior step. Final DNA concentration was measured using the Qubit 3.0 High Sensitivity range assay (Fisher Scientific, Ottawa, ON, Canada). The purity of the DNA was evaluated using a Nanodrop ND-1000 Spectrophotometer (Thermo Scientific, Wilmington, DE, USA) and by gel electrophoresis (3% of agarose). The amplicons were then sent to McGill University and Genome Quebec Innovation Center (Montreal, QC, Canada) for purification, barcoding and sequencing by the Illumina MiSeq 250 paired-ends sequencing system. The sequences were trimmed with fqCleaner v.3.0 and assembled with SPAdes v.3.11. Assembly quality was assessed using the number of contigs N50 and L50 metrics. 

### 4.6. MLST and cgMLST Characterization and Virulence, Antimicrobial Resistance and Stress-Related Genes 

The BLASTN algorithm [13,75] was used to extract the cgMLST profiles (1748 loci; [13]). The profiles were grouped into sequence types (ST) and clonal complexes (CCs) [76]. As previously described by Moura et al. (2016), cut-offs of 7 and 150 allelic mismatches were used, respectively, in order to group the isolates profiles into cgMLST types (CTs) and sublineages (SLs). The BIGSdb-Lm platform was used for the identification of virulence, antimicrobial resistance, and stress-related genes. The dendrogram was built on BioNumerics v.7.6.3 using the single linkage clustering algorithm [13].4.7.

### 4.7. DNA Extraction and Purification of the Total Microbiota

The total DNA of the pool of the two pellets of each sample was extracted and purified using a modified version of a phenol-chloroform protocol as described in Larivière-Gauthier et al. (2017) [77]. Briefly, 350 μL of lysis buffer (500 mM Tris-HCl, 200 mM EDTA, 1% SDS (*w*/*v*), Fisher Scientific, Ottawa, ON, Canada) was added to each pellet to resuspend them, to allow their pooling and to perform a chemical lysis. The mixed solution (700 μL) was then added in microtubes containing 0.1 mm glass beads (MP Biomedicals, OH, USA). A cell mechanical lysis was performed using a MP-Fastprep-24 5GTM High-Speed Homogenizer (MP Biomedicals, Santa Ana, CA, USA) twice at an intensity of 6.0 m/s for 40 s. Samples were kept for five minutes on ice between cycles. DNA purification was conducted using a standard phenol/chloroform protocol [78]. Final DNA concentration was measured using the Qubit 3.0 High Sensitivity range assay (Fisher Scientific, Ottawa, ON, Canada). The purity of the DNA was evaluated using a Nanodrop ND-1000 Spectrophotometer (Thermo Scientific, Wilmington, DE, USA) and by gel electrophoresis (3% of agarose). The six negative experimental controls were processed in parallel with the samples as well as the negative DNA extraction controls that consisted of a 700 μL lysis buffer without bacterial pellets. Purified DNA samples were stored at −80 °C until sequenced.

### 4.8. Total Microbiota 16S Sequencing and Bio-Informatics Analyses 

A 292 bp segment of the V4 hypervariable region of the 16S RNAr gene was amplified using universal primers targeting the total bacterial and archaeal populations (515F_Ill and 806R_Ill, Invitrogen, Thermo Fisher Scientific, Waltham, MA, USA) [79,80]. A 30 µL PCR reaction was carried out using the Platinum SuperFi PCR Master Mix (Invitrogen, Burlington, ON, Canada). Ten nanograms of DNA from each sample were amplified for 27 cycles with a denaturation step at 98 °C for 30 s, an annealing step at 55 °C for 30 s, an elongation step at 72 °C for 180 s and a final elongation step of 10 min at 72 °C. One microlitre of an artificial community (ZymoBIOMICS Microbial Community DNA Standard) (Zymo Research, Irvine, CA, USA) was diluted in 10 μL of sterile water to serve as a positive control and as an indicator of the quality of the sequencing. Five positive controls were integrated to the PCR plates to evaluate the reproducibility of the results. Experimental controls as well as negative extraction controls and negative PCR controls were also added to the plates. The amplification of the DNA target segment and the absence of amplification from the negative controls were validated by gel electrophoresis (3% of agarose). The amplicons were then sent to McGill University and Genome Quebec Innovation Center (Montreal, QC, Canada) for purification, barcoding and sequencing by Illumina MiSeq 250 paired-ends sequencing. 

The cleaning and the analyzing of the sequences were completed using Mothur 1.39.5 according to Larivière-Gauthier and al. (2017) [77]. The primers were first removed and then the complementary sets of reads were merged for each sample. Sequences that contained ambiguities were removed and identical sequences were grouped. The sequences were then aligned using the SILVA database V132. The chimeras were removed using UCHIME. The remaining sequences that were similar at 97% were grouped into operation taxonomic units (OTUs) using the PDS database (Trainset16). 

Alpha and beta diversity analysis were performed using RStudio 3.6.1. The lowest number of sequences in the samples was used as a subsampling. For the alpha diversity, the coverage of the subsampling was measured as well as the number of OTUs in each sample and their evenness using the inverse Simpson and the Shannon indices. Comparison statistics were performed between the *L. monocytogenes*-positive samples and the *L. monocytogenes*-negative samples using Student’s t-test with a significance level of 0.05. For the beta diversity, Jaccard and Bray–Curtis dissimilarity indices were used on the same subsampling. Non-metric multidimensional scaling graphs (NMDS) were used for the visualization of the results. An ANOVA was used to compare the beta diversity of the positive and negative samples for *L. monocytogenes* with a significance level of 0.05. In addition, the Multivariate Association with Linear Models method (MaAsLin version 1) was used to identify OTUs significantly associated with the detection or the absence of *L. monocytogenes* in terms of relative abundance.

## 5. Conclusions

The aims of our study were to (i) characterize the genomic diversity of *Listeria monocytogenes* isolates harvested from the six conveyor belts of the cutting room of a swine slaughterhouse, (ii) to evaluate the heterogeneity of the spatial and temporal contamination of these surfaces by the pathogen and (iii) to identify, using 16S rRNA amplicon sequencing, microbial determinants of the presence or absence of the bacteria. We were able to identify a low genomic diversity, to anticipate an attenuation of the virulence into our isolates as well as highlight important characteristics of persistence. We also identified a clear preferential localization of *Listeria monocytogenes* on three conveyor belts, thus posing the hypothesis of a potential role of the background microbiota associated with the different meat cuts in the sheltering of *Listeria monocytogenes* on certain preferential sites. We also identified, using an original HTS approach and for the first time to our knowledge, a positive interaction between the taxa *Veillonella* (OTU00380) and the presence of *Listeria monocytogenes* on food-contact surfaces. The interaction is currently being studied in the laboratory. We believe that our model for studying the relationship between the accompanying microbiota and *Listeria monocytogenes* represents a step towards a more realistic and complex approach to the presence of the pathogen in the food industry. We think that a better understanding of the composition of the microbial environment around *Listeria monocytogenes* could lead to an approach where the niches allowing the growth of the pathogen can be targeted in the food processing environment.

## Figures and Tables

**Table 1:** Comparison of alpha-diversity indices of the positive *Listeria monocytogenes* samples’ microbiota and the negative *Listeria monocytogenes* samples’ microbiota.

Table.	Presence	Absence
Observed	230.88	223.07
Shannon	3.13	3.24
Inv. Simpson	11.63	12.74

The means were based on 1000 subsampling of 10,087 sequences. Absence: negative *Listeria monocytogenes* microbiota in samples, Presence: positive *Listeria monocytogenes* microbiota in samples.

## Data Availability

The data presented in this study are openly available in the NCBI Sequence Read Archive under accession number PRJNA758928 (https://www.ncbi.nlm.nih.gov/sra/PRJNA758928, accessed on 1 September 2021).

## Supplementary Materials

The following are available online at https://www.mdpi.com/article/10.3390/pathogens10111368/s1, Table S1: Preliminary characterization of *Listeria monocytogenes* isolates, Figure S1: Alpha diversity comparison of the positive *Listeria monocytogenes* samples’ microbiota and the negative *Listeria monocytogenes* samples’ microbiota, Figure S2: Non-Metric Multidimensional Scaling Graph (NMDS) of the microbiota structures of the *Listeria monocytogenes* positive samples and the *Listeria monocytogenes* negative samples using the Jaccard index.
