# Supplementary material for: Use of an Ecosystem-Based Approach to Shed Light on the Heterogeneity of the Contamination Pattern of Listeria monocytogenes on Conveyor Belt Surfaces in a Swine Slaughterhouse in the Province of Quebec, Canada
Source: Pathogens. 2021 Oct 22;10(11):1368. doi: 10.3390/pathogens10111368 (PMC8625388; doi:10.3390/pathogens10111368)
Supplement: Supplementary file 1 [file pathogens-10-01368-s001.zip › pathogens-1392563-supplementary.pdf]

Table S1. Preliminary characterization of *Listeria monocytogenes* isolates.

Isolate ID	Serotype	InlA type	Biofilm type	
			12°C	30°C
V2CP1A	1/2a	Truncated	Weak	Weak
V2CP1B	3a	Truncated	Strong	Strong
V2CP2A	3c	Complete	Weak	Weak
V2CP2B	3c	Complete	Weak	Weak
V2CP3A	1/2c	Truncated	Moderate	Moderate
V2CP3B	1/2c	Truncated	Moderate	Moderate
V2CP5A	1/2c	Truncated	Moderate	Moderate
V2CP5B	1/2c	Truncated	Moderate	Moderate
V2BO1A	3c	Complete	Weak	Weak
V2BO1B	3c	Complete	Weak	Weak
V2BO2A	1/2a	Complete	Moderate	Moderate
V2BO2B	1/2a	Truncated	Strong	Strong
V2BO4A	1/2c	Truncated	Moderate	Strong
V2BO4B	1/2c	Truncated	Moderate	Moderate
V2BO5A	3a	Complete	Weak	Weak
V2BO5B	3a	Complete	Weak	Weak
V2PI4A	1/2a	Truncated	Weak	Moderate
V2PI4B	3a	Truncated	Moderate	Weak
V3CP3A	1/2a	Truncated	Strong	Moderate
V3CP3B	1/2a	Truncated	Moderate	Strong
V3CP5A	1/2a	Truncated	Moderate	Weak
V3CP5B	1/2a	Truncated	Moderate	Moderate
V3CP6A	1/2a	Truncated	Moderate	Weak
V3CP6B	1/2a	Truncated	Weak	Moderate
V3CP7A	1/2a	Truncated	Strong	Moderate
V3CP7B	3a	Truncated	Strong	Moderate
V3CP8A	1-2a	Truncated	Moderate	Strong
V3CP8B	1/2a	Truncated	Strong	Strong
V3BO1A	3a	Truncated	Strong	Moderate
V3BO1B	3a	Truncated	Moderate	Strong
V3BO3A	1/2a	Truncated	Strong	Strong
V3BO3B	1/2a	Truncated	Moderate	Strong
V3BO4A	1/2a	Truncated	Strong	Moderate
V3BO4B	3a	Truncated	Weak	Weak
V3BO5A	1/2a	Truncated	Weak	Strong
V3BO5B	1/2a	Truncated	Strong	Strong
V3BO8A	1/2a	Truncated	Moderate	Strong
V3BO8B	1/2a	Truncated	Strong	Strong
V3PI2A	1/2a	Truncated	Moderate	Moderate

V3PI2B	1/2a	Truncated	Moderate	Strong
V3PI3A	1/2a	Truncated	Moderate	Moderate
V3PI3B	1/2a	Truncated	Moderate	Moderate
V4CP1A	1/2a	Complete	Weak	Moderate
V4CP1B	1/2a	Complete	Weak	Weak
V4CP6A	1/2a	Truncated	Strong	Moderate
V4CP6B	1/2a	Truncated	Strong	Moderate
V4CP7A	1/2a	Truncated	Weak	Moderate
V4CP7B	1/2a	Truncated	Weak	Weak
V4BO6A	1/2a	Truncated	Moderate	Moderate
V4BO6B	1/2a	Truncated	Moderate	Moderate
V4PI1A	1/2a	Truncated	Moderate	Weak
V4PI1B	1/2a	Truncated	Moderate	Moderate
V5BO1A	1/2a	Truncated	Moderate	Weak
V5BO1B	1/2a	Truncated	Weak	Moderate
V5BO2A	1/2c	Truncated	Moderate	Moderate
V5BO2B	1/2c	Truncated	Moderate	Moderate
V5PI1A	1/2c	Truncated	Moderate	Strong
V5PI1B	1/2c	Truncated	Strong	Moderate
V5PI2A	1/2a	Truncated	Moderate	Moderate
V5PI2B	1/2a	Truncated	Strong	Moderate
V5PI4A	1/2c	Truncated	Moderate	Moderate
V5PI4B	1/2c	Truncated	Strong	Strong
V5PI6A	1/2a	Truncated	Weak	Weak
V5PI6B	1/2a	Truncated	Strong	Strong
V6BO1A	1/2a	Truncated	Weak	Weak
V6BO1B	1/2a	Truncated	Strong	Weak
V6BO3A	1/2a	Truncated	Moderate	Moderate
V6BO3B	1/2a	Truncated	Moderate	Strong
V6PI2A	1/2b	Truncated	Moderate	Weak
V6PI2B	1/2b	Truncated	Weak	Weak
V6PI6A	1/2b	Complete	Moderate	Weak
V6PI6B	1/2c	Truncated	Moderate	Weak

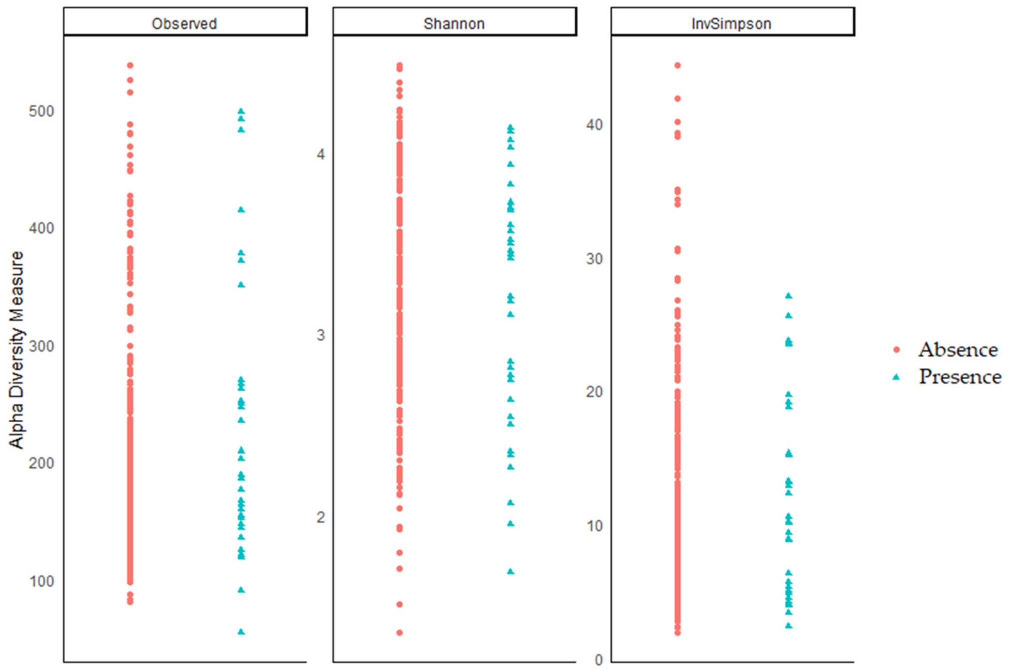

Figure S1. Alpha diversity comparison of the positive Listeria monocytogenes samples microbiota and the negative Listeria monocytogenes samples microbiota.

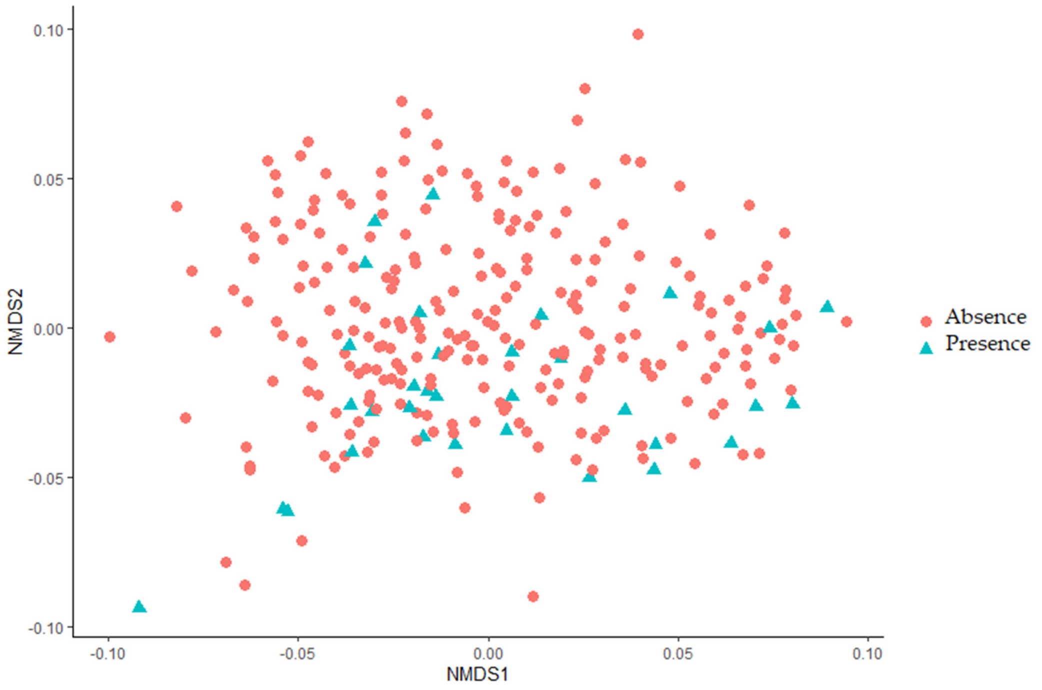

Figure S2. Non-Metric Multidimensional Scaling Graph (NMDS) of the microbiota structures of the Listeria monocytogenes positive samples and the Listeria monocytogenes negative samples using the Jaccard index.
